# “Who am I without my disorder?”: An interpretative phenomenological analysis of recovery from combined bulimia nervosa and borderline personality disorder

**DOI:** 10.1186/s40337-026-01716-5

**Published:** 2026-08-13

**Authors:** Gemma Turner, Maike Klein

**Affiliations:** Division of Health Research, Lancaster University, Health Innovation Campus, 1 Sir John Fisher Drive, Lancaster, LA1 4YW UK

**Keywords:** Bulimia nervosa, Borderline personality disorder, Eating disorder, Recovery, Treatment, Lived experience, Interpretative phenomenological analysis

## Abstract

**Background:**

Co-occurring Bulimia Nervosa (BN) and Borderline Personality Disorder (BPD) affect a substantial population in the United Kingdom. This study explored how individuals experience recovery from these conditions, with a focus on interpreting how individuals with such experiences make sense of themselves.

**Methods:**

Semi-structured interviews were conducted with twelve individuals with lived experience of recovery from BN and BPD. Data were analysed using Interpretative Phenomenological Analysis.

**Results:**

Three superordinate themes were identified: (1) Ambivalence around recovery, reflecting conflicting messages from care systems; (2) Controlled chaos and support, capturing emotional volatility, relational instability, and the non-linear nature of recovery; and (3) Identity, stigma, and the search for self, highlighting developing self-compassion, agency, and identity shifts. Recovery was described as an evolving, relational process shaped by ambivalence and self-redefinition. Therapeutic trust and authentic connection facilitated recovery, while invalidating environments, trauma, and service limitations acted as barriers.

**Conclusions:**

Recovery from co-occurring BN and BPD is a complex, relational process involving identity reconstruction and emotional integration. Findings suggest that recovery-oriented services should prioritise compassionate, trauma-informed, and relationally attuned care, with particular attention to reducing diagnostic stigma, fostering collaborative therapeutic relationships, and supporting the development of a meaningful sense of self beyond illness.

## Abstract (articlehighlights)

Bulimia nervosa (an eating disorder) and borderline personality disorder (a condition linked to intense emotions and relationship difficulties) often occur together. However, we know very little about how people experience recovery when living with both conditions. This study explored the personal experiences of 12 people in the UK who described themselves as being in recovery. Participants shared that recovery is not a simple or straightforward process. Instead, it is often confusing, emotional, and involves both progress and setbacks. Many described feeling unsure about who they were without their difficulties, as these had become part of their identity over time. Letting go of these behaviours could feel both hopeful and frightening. Supportive relationships, especially with therapists, friends, and family, were seen as very important. Feeling understood, accepted, and not judged helped people move forward. In contrast, negative or dismissive experiences within services made recovery harder. Overall, recovery was described as a gradual process of rebuilding a sense of self, learning to cope with emotions in new ways, and developing self-compassion.

## Background

Co-occurring Bulimia Nervosa (BN) and Borderline Personality Disorder (BPD) affect an estimated 900,000–1.2 million people worldwide, with over 300,000 individuals seeking treatment annually in the United Kingdom (UK; [1, 2]). This overlap is substantial, with around 25% of individuals with BN meeting criteria for BPD, and elevated rates of BN among those diagnosed with BPD [3, 4]. More commonly reported among women and frequently linked to trauma and adverse childhood experiences [5, 6], this combination reflects shared features such as emotional dysregulation, impulsivity, and maladaptive coping strategies [7, 8]. These overlapping characteristics can reinforce symptom severity and increase distress, self-harm, and suicidality [9]. In addition to their clinical complexity, both BN and BPD are associated with considerable stigma. Individuals with BN often report feeling misunderstood or overlooked, particularly when they do not fit stereotypical expectations of eating disorders, while BPD has been described as one of the most stigmatised mental health diagnoses within healthcare settings [10]. Such stigma may affect willingness to seek help, disclose symptoms, or engage with services, and may influence how individuals are perceived and treated by healthcare professionals. For individuals experiencing both BN and BPD, these challenges may create additional barriers to accessing appropriate care and support.

Effective treatment therefore requires flexible, trauma-informed approaches that address behavioural, emotional, and relational dimensions simultaneously [11]. Cognitive Behavioural Therapy (CBT) remains the recommended first-line treatment for BN, while Dialectical Behaviour Therapy (DBT) has demonstrated benefits for individuals with BPD and has increasingly been applied within eating disorder services [12, 13]. However, individuals with co-occurring BN and BPD often present with greater clinical complexity, higher levels of psychopathology, and poorer treatment outcomes than those without BPD [14, 15]. Emerging evidence has therefore highlighted the potential value of integrated approaches that simultaneously address eating disorder and BPD-related difficulties, although the evidence base remains relatively limited [4].

While therapies such as CBT and DBT show promise in contributing to symptom reduction, one cannot underestimate the systemic barriers which individuals with co-occurring BN and BPD frequently experience, such as challenges engaging with treatment due to the complexity of their difficulties, interpersonal mistrust, and experiences of stigma [10, 16–18]. Such factors may limit access to care, contribute to treatment discontinuation, and complicate recovery trajectories. Traditionally, treatment outcomes in both eating disorders and personality disorders have been evaluated primarily through symptom reduction and behaviour change. However, recovery-oriented frameworks have increasingly challenged this perspective, proposing that recovery encompasses more than absence of symptoms [19]. Instead, recovery is being understood as a personal and non-linear process involving identity reconstruction, self-acceptance, emotional resilience, emotional wellbeing, fulfilling relationships, even in the presence of ongoing difficulties [19–21]. Recovery-oriented frameworks therefore place emphasis on individuals' subjective experiences and understandings of recovery alongside clinical outcomes.

While a substantial body of qualitative literature has explored the lived experiences of eating disorder recovery more broadly (e.g., [22–25]), little is known about how recovery is experienced among individuals with co-occurring BN and BPD, whose experiences may be shaped by additional challenges relating to emotional dysregulation, interpersonal difficulties, identity disturbance, and stigma. Given these complexities, it remains unclear whether existing recovery frameworks adequately capture the experiences and priorities of this population. Developing a deeper understanding of these experiences may help refine existing recovery frameworks, inform more integrated and trauma-informed approaches to care, and ensure that treatment models better reflect the needs and priorities of individuals navigating recovery from both conditions. Therefore, this study aims to generate an in-depth understanding of how individuals with co-occurring BN and BPD experience and make sense of recovery.

## Methods

### Design

This study sought to address the following questions:

1. What are the lived experiences of recovery among individuals with combined BN and BPD?
2. How do individuals make sense of their recovery from, and identity with, BN and BPD, in light of these experiences?

Although a substantial body of qualitative research has explored eating disorder recovery more broadly, little is known about how individuals experience recovery from co-occurring BN and BPD, or how they make sense of themselves in relation to these experiences. Given the study's focus on exploring the meaning participants attribute to recovery and identity reconstruction, Interpretative Phenomenological Analysis (IPA; [26]) was selected as the most appropriate methodology. Grounded in phenomenology, hermeneutics, and ideography, IPA is particularly well suited to exploring how individuals understand and make sense of significant life experiences. It therefore offers a valuable framework for exploring individuals’ lived experiences, interpreting the meaning of these, and emphasising the individual over the whole population experience [27].

### Sample and recruitment

In line with IPA, samples should be sufficiently homogenous in relation to the phenomenon under investigation, enabling an in-depth exploration of shared lived experiences rather than comparison across substantially different groups [28]. In the present study, the phenomenon of interest was the lived experience of recovery from combined BN and BPD-related difficulties. Accordingly, inclusion criteria required participants to be over 18 years of age, have a previous formal diagnosis of BN, and either a formal diagnosis or self-identify with BPD. Participants also needed to self-identify as being in recovery, or be considered in recovery by a professional, must have been out of treatment for a minimum of three months, and be a peer support worker or ambassador affiliated with the participating eating disorder charity. Exclusion criteria included individuals under 18 years, those currently receiving treatment for BN or BPD, those who had relapsed within the past three months, those without a formal diagnosis of BN, and those who had neither a formal diagnosis nor self-identification with BPD.

Eligibility was established through participant verbal report during screening. Participants confirmed that they had previously received a formal diagnosis of BN from a psychiatrist within a specialist eating disorder service. Information regarding BPD was also obtained through participant report and reflected either a previous formal diagnosis or self-identification. Diagnostic status was not independently verified through clinical records. Although participants differed as to whether their BPD was formally diagnosed or self-identified, all were judged to share the experience of having recovered from difficulties associated with BN and BPD.

Recruitment followed a purposive sampling strategy and was facilitated through a UK-based eating disorder charity, which advertised the study in its national volunteer newsletter. Participation in this study was specifically limited to peer support workers and ambassadors affiliated with the charity, rather than the broader population of individuals recovering from BN and BPD. This approach was informed by ethical considerations, as ambassadors and volunteers have access to established support structures to manage any distress arising from participation. In addition to the research being recovery-focused, this criterion helped minimise potential risk of harm. Interested individuals contacted the researcher directly and participated in a brief eligibility screening. Those meeting the inclusion criteria were formally invited to participate and provided with a detailed.

information sheet, outlining the study’s aims, participant involvement and procedures related to ethics, such as confidentiality and their right to withdraw. Twelve participants (one man, 11 women) took part in the study. A summary of participant demographics can be found in Table 1.

**Table 1:** Summary of participant characteristics

Pseudonym	Gender	Age	Location	Ethnicity	BN formal diagnosis	BPD formal diagnosis or self-identified
Hayley	Female	24	NW England	White British	Yes	Formal
Connor	Male	28	South England	White British	Yes	Formal
Rebecca	Female	37	NW England	Asian	Yes	Formal
Laura	Female	29	South England	White British	Yes	Formal
Kacey	Female	65	SE England	White British	Yes	Self-Identified
Georgia	Female	42	North England	White British	Yes	Self-Identified
Sarah	Female	25	West England	White British	Yes	Formal
Vienna	Female	36	NW England	White British	Yes	Formal
Leyla	Female	35	NW England	White British	Yes	Self-Identified
Emery	Female	47	North England	White British	Yes	Formal
Emalia	Female	32	NW England	White British	Yes	Formal
Kerry	Female	44	NW England	White British	Yes	Self-Identified

### Data generation

Data were generated through individual, semi-structured interviews, which were offered online, via Microsoft Teams, and lasted for approximately 90 min. These were audio-recorded, using a password-protected recording device, and subsequently uploaded to a folder within a secure cloud storage system. Each interview was then transcribed verbatim and anonymised, using pseudonyms. Upon completion of transcription, these recordings were securely destroyed. Interviews were flexible yet focused on exploring participants' recovery experiences from BN and BPD, including their understandings of recovery, perceived facilitators and barriers to recovery, experiences of treatment and support services, the impact of recovery on their sense of identity, and how they made sense of themselves in relation to their recovery journeys. While guided by a set of core questions, the format allowed participants to share their stories in their own words and raise topics of personal significance. Example questions included: "Can you tell me about your experiences of recovery from BN and BPD?", and "How, if at all, has recovery influenced how you see yourself?". The full interview schedule is available from the corresponding author upon reasonable request. The interview guide was reviewed by an expert by experience, prior to commencing the interview phase of the project, to ensure appropriateness and accessibility of language. Interviews were adapted responsively to participants’ needs and cues. Ethical considerations were rigorously observed: participants gave informed written consent prior to interview and reiterated verbal consent on commencing the interviews. All were assured of confidentiality and could withdraw at any time up until programme completion. Given the sensitive nature of the topics discussed, time to debrief after the interview was provided, with the recording device switched off. Participants were also provided a debrief sheet, outlining relevant resources.

### Ethics

This study received ethical approval from the host University’s Faculty for Health and Medicine Research Ethics Committee.

### Reflexivity

This research was conducted by a trainee clinical psychologist and supervised by a counselling psychologist and consultant clinical psychologist, all with expertise in the area of eating disorders and complex trauma. These professional backgrounds shaped the analytic process by sensitising the research team to the emotional, relational, and systemic dimensions of participants’ experiences. In particular, the researchers were attuned to themes relating to trauma, identity, emotional regulation, stigma, and therapeutic relationships, given their relevance within both clinical practice and the existing literature. Reflexivity was understood not solely as the management of bias, but as an ongoing process of critically examining how the researchers’ perspectives informed interpretation. Throughout the study, the first author maintained a reflective journal [29] to document assumptions, emotional responses, and developing interpretations. These reflections were discussed regularly within supervision with the second author, where alternative interpretations were explored and emerging themes critically examined. Such discussions occasionally challenged initial assumptions and encouraged greater attention to participants’ own meaning-making, enriching the depth and nuance of the analysis. Rather than attempting to eliminate the influence of the researcher, reflexive practices were used to promote transparency and support a thoughtful, interpretative engagement with the data.

### Data analysis

Data analysis followed the six steps of IPA, as outlined by Smith and colleagues (2009). These are: (1) Reading and re-reading, (2) Initial noting, (3) Developing emergent themes, (4) Searching for connections across themes, (5) Moving to the next case, and (6) Looking for patterns across cases. In the first step, each transcript was reviewed multiple times, to familiarize with the data and promote bracketing; the process of noting down the researcher’s initial thoughts, presumptions and biases, and to set these aside (i.e., bracket) before any further analysis. The second step involved line-by-line coding descriptive, linguistic, and conceptual comments relating to participants’ felt and embodied experiences as well as their way of making sense of their experiences. This process is also referred to as double hermeneutics [28], which means the researcher attempts to make sense of the participants making sense of their experiences, language, emotions, and meaning making. The third step involved connecting these codes and identifying patterns relating to commonalities or differences. Step four then involved grouping these themes into superordinate themes. In line with IPA’s idiographic nature, step five involved repeating these previous four steps for each subsequent transcript before moving onto the final step, whereby patterns and themes across participants are captured. Data was recorded and tracked on a Microsoft Excel spreadsheet.

## Results

**Fig. 1:**
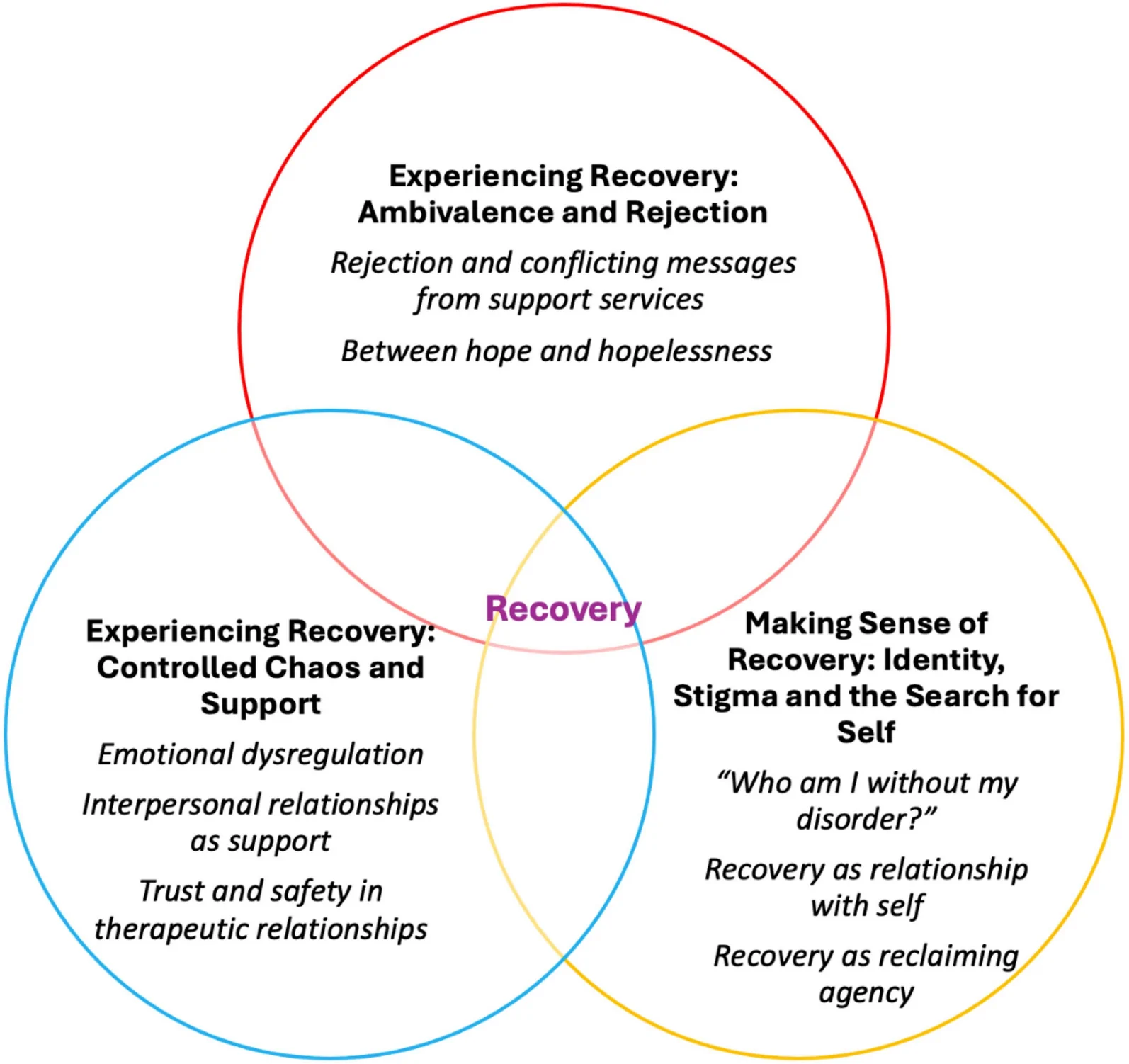
Relationship between Superordinate and Sub-Themes

The analysis generated three superordinate themes, all relating to participants’ lived experiences of recovery from combined BN and BPD. Theme one ‘Ambivalence and Rejection’ and theme two ‘Controlled Chaos and Support’ together address the first research question, whilst theme three ‘Identity, Stigma and the Search for Self’ directly addresses the second research question. This section now presents each theme with the support of anonymized participant quotes. The relationship between these themes is illustrated in Fig. 1, offering further depth to the analytical commentary.

### Experiencing recovery: ambivalence and rejection

This superordinate theme was shared by all 12 participants and highlights participants complex feelings of ambivalence towards recovery, often reinforced by inconsistent or conflicting messages from healthcare systems. It is depicted in two sub-themes, presented below.

#### Rejection and conflicting messages from support services

Participants described a recurring and distressing pattern in their engagement with healthcare services, in which access and continuation of treatment were often contingent on weight or observable behavioural change. Nine participants shared the belief that they had to *“get worse to get better”,* feeling that their suffering was only taken seriously when their weight reached a critical low. This reinforced not only disordered beliefs around worthiness and illness but also entrenched the idea that visible deterioration was a necessary gateway to care. Seeking help was often prompted by external pressures or the threat of losing something important, such as employment or a valued relationship. However, attempts to access specialist services were frequently met with rejection, particularly when participants were not underweight. This reinforced a damaging message that their suffering was not *“valid enough”*, intensifying feelings of unworthiness and perpetuating the belief that they needed to deteriorate further to be taken seriously. Eleven participants articulated this belief. For example, Vienna shares:

“I was saying I was struggling with food, and it felt like they were saying ‘well you don’t look like you are’, or ‘well you’re not struggling enough’. I felt like I needed to lose weight before I deserved support to get better.”

For those who did access services, progress frequently led to premature discharge, often before participants felt psychologically ready. Conversely, if participants struggled to make measurable behavioural improvements, they were also discharged on the grounds that treatment was *“not working”*, as expressed by Vienna:

“If I made changes people, clinicians, staff would assume that I was feeling better and talk about discharge. If I didn’t make changes they said I wasn’t engaging and would talk about discharge. Either way I didn’t feel OK.”

These experiences of invalidation, misunderstanding, and rejection from healthcare professionals became central to participants’ narratives of recovery, echoing earlier experiences of relational neglect and trauma. A particularly salient theme was the misalignment between service priorities and participant needs: while services emphasised weight stabilisation or behavioural change, participants felt these outcomes did not address their core emotional distress or the underlying trauma that fuelled their disorders. The assumption that behavioural improvement equated to recovery often left participants feeling abandoned, unseen, and emotionally unresolved, further complicating their path toward healing, as expressed by Emery:

“I get why they had to think about behavioural change, but how can they expect me to give up my coping strategy without dealing with the issue that warranted my coping strategy?”

Participants described a long-standing sense of silence and suppression surrounding their traumatic experiences, particularly those involving interpersonal and relational distress. Eleven shared that throughout their contact with mental health services, the focus remained on symptom management, such as eating behaviours or emotional instability, with little or no attention given to the origins of their distress. For instance, Georgia shares: *“They kept asking what was wrong with me, but no one ever asked what happened to me.”* This absence of inquiry left many feeling misunderstood and emotionally neglected, compounding their sense of isolation. For nine of those 11, recovery began with the reclaiming of their voice; the act of being heard, validated, and allowed to speak about their trauma without fear of dismissal or judgment. Sharing their story became not only a therapeutic turning point but also an act of resistance against a system that had historically silenced them, as expressed by Sarah:

“I remember the first time I talk about what has happened to me. It was like a weight had been lifted. My therapist was really validating and she kept saying it wasn’t my fault. That was the first time I remember thinking that actually, maybe I didn’t deserve my eating disorder.”

The process of voicing trauma was often described as painful but necessary, central to developing a more coherent sense of self and beginning to heal the fractures in their identity caused by chronic invalidation and unacknowledged suffering.

### Between hope and hopelessness

Participants described recovery as a deeply fluctuating and emotionally ambivalent process, marked by alternating states of hope and despair. On some days, individuals felt that recovery was within reach, expressing a sense of possibility and motivation for change. Yet on other days, this sense of progress was overshadowed by chronic emotional pain, self-doubt, and a pervasive fear of relapse, as expressed by Sarah:

“There were days when I felt like I could really do this. Like, really beat the illness. But then other days I just thought, this is how it’ll be for the rest of my life.”

These oscillations in belief and emotional state were not only intrinsic to their internal struggles but were also shaped by external experiences of stigma, particularly from healthcare professionals. Participants recalled moments where their distress was minimised, dismissed, or misunderstood, which echoed earlier experiences of relational trauma and reinforced feelings of worthlessness and hopelessness. For instance, Connor shares:

“Sometimes people were quite harsh, like about things I was experiencing. Like saying ‘just get better’ or ‘you’re doing this on purpose’ or whatever. You know, making you feel bad for doing certain things.”

In summary, in the context of their BN and BPD diagnoses, participants described feeling that the emotional distress associated with BPD was frequently dismissed as attention-seeking or self-inflicted, while the seriousness of BN was sometimes overlooked when they did not conform to stereotypical expectations of eating disorder presentations. Participants experienced this combination as leaving them feeling misunderstood and unseen, reinforcing doubts about whether recovery was achievable.

### Experiencing recovery: controlled chaos and support

This superordinate theme was shared by all 12 participants and highlights the navigation of complex emotions, interpersonal turmoil and non-linear process of healing. It is depicted in three sub-themes, which are presented below.

#### Fragile recovery: navigating the emotional complexity of healing

Eight participants described recovery not as a clean break from illness, but as a complex, ongoing negotiation with distress, identity, and coping. Even when BN behaviours subsided, many reported that the emotional volatility associated with BPD persisted, often more acutely felt without the buffering effects of disordered eating. This led to a sense that recovery, rather than bringing relief, sometimes exposed them more directly to emotional pain, intensifying feelings of vulnerability. The loss of BN as a coping mechanism, while necessary for healing, also created a void, where participants struggled to contain or regulate emotional states that remained deeply unstable. For example, Sarah expresses: *“I felt like, how am I supposed to cope with everything I had going on without my ED? It had become my best friend that I’d known for over 10 years”*. Participants often described BN as a means of regulating the intense emotional experiences associated with BPD. As eating disorder behaviours reduced, many felt increasingly exposed to emotional pain, interpersonal sensitivity, and identity instability. Recovery therefore involved not only relinquishing eating disorder behaviours but also learning to navigate enduring BPD-related difficulties without a familiar coping strategy.

The process of recovery was consistently described as non-linear, with five participants rejecting the idea of a final, stable *“recovered”* state. Instead, recovery was experienced as cyclical, fragile, and constantly shifting, marked by both setbacks and progress. Some framed recovery as the maintenance of gains rather than the achievement of a complete transformation, as shared by Emalia: *“I don’t think I’ll ever be fully free from [BN and BPD]. I think I’ll always have to choose to make good choices, maybe even on a daily basis.”* This ambivalence reflected the realities of living with dual diagnoses, where emotional intensity, relational sensitivity, and identity instability could resurface at any time. Additionally, participants described their ED as a way to create order amidst emotional and environmental turbulence. BN behaviours provided a predictable and private form of control, especially when other aspects of life, such as trauma histories, interpersonal relationships, or internal states, felt overwhelming or uncontrollable. For example, Leyla highlights:

“I remember sitting there and watching my entire world fall apart around me. Then I looked down at my body and just thought ‘I can control this’. That’s when it all started.”

Letting go of these behaviours in recovery felt, for some, like letting go of the only stability they had ever known. The resulting tension between the desire to heal and the fear of being emotionally overwhelmed, highlighted the profound ambivalence many participants felt about recovery, as well as the need for approaches that address both behavioural symptoms and the enduring emotional pain that underpins them.

#### Interpersonal relationships as support

A recurring theme within participants’ narratives was the tension between a deep longing for connection and a profound fear of relational pain. Ten participants described a tendency to push others away, even when support was desperately needed, often rooted in earlier experiences of abandonment, abuse, or inconsistent caregiving. Connor highlights:

“I just so badly wanted someone to care for me and help me. But I couldn’t open up because I thought that they would hate me for being so miserable and weird. So I backed away and isolated myself before they got chance to reject me”.

This paradox was closely linked to both BN and BPD, where patterns of attachment insecurity, shame, and relational instability shaped how participants engaged with others and with recovery itself. Participants often located the origins of their distress in early disconnection or exclusion, and this enduring sense of not belonging remained central in both the maintenance of their disorders and the obstacles they faced in recovery. The BN, for some, became a substitute for emotional connection; a reliable presence when human relationships felt unsafe or unavailable. Georgia shares:

“I wanted to escape [my ED] but at the same time, it was like my best friend. It was predictable when people weren’t. It was reliable when people weren’t. My ED was my safe place to turn when life was painful.”

This dynamic created ambivalence toward recovery, particularly when it was a significant relationship, such as a friend or partner that first noticed their distress and encouraged help-seeking. Recovery was not simply about giving up disordered behaviours, but about relinquishing the only sense of belonging some participants had ever known.

“My best friend came with me to a support group, and I was so grateful because she was so supportive and so kind and she didn’t judge me at all. But the constant fear that she would ditch me at some point, made it really hard to actually want to give up my ED (…) My ED was the only thing that had always been there” (Leyla)

Nonetheless, all 12 participants identified turning points in recovery as moments of genuine interpersonal connection: experiencing love, acceptance, or being *“seen”* without judgment. Feeling part of something, whether through friendship, family, work, or community, was described as a transformative factor that allowed participants to begin developing reciprocal, secure relationships that eventually rendered the disorder redundant. For instance, Vienna expresses:

“I started a new job after being off sick for ages and for the first time ever, I started to think, ‘I can do this’. And people were really kind and supportive. They just seemed to accept me for who I was. It felt weird at first but now I can’t imagine my life without those friends. Real, true friends”

Conversely, interpersonal stress, feeling misunderstood, excluded, or different, was a common trigger, often driving efforts to control weight, shape, or food in a quest for acceptance. Kacey shares: *“If I lost weight, they’d like me more. Rationally, I know it doesn’t make sense, but I really genuinely believed that, and sometimes I still have to fight those thoughts now.”* Recovery narratives frequently included a shift from external validation to internal acceptance, marked by a move away from striving for perfection through appearance or performance. Instead, participants spoke of learning to value self-acceptance, body acceptance, and being accepted by others not for how they looked or behaved, but for who they were. This underscores the profound relational underpinnings of both disorder and recovery, highlighting that healing is often made possible through connection, belonging, and the slow rebuilding of trust in oneself and others.

#### Trust and safety in therapeutic relationships

For participants navigating recovery from both BN and BPD, the therapeutic alliance emerged as a pivotal component of their recovery journey. Given the profound difficulties with trust, fear of rejection, and sensitivity to invalidation often associated with BPD, participants described the process of opening up in therapy as slow, fragile, and deeply contingent on the relationship with the professional. Trust had to be earned, not assumed.

All 12 participants shared that they could only begin to engage in meaningful therapeutic work once they felt truly seen, heard, and accepted without judgment. For example, Kacey expresses:

“It took me quite a few sessions to really open up. You can’t just talk about trauma, you know, you have to really be able to trust someone because you feel vulnerable.”

Notably, participants placed far less emphasis on the professional’s role, qualifications, or clinical experience, and far more on their relational qualities. For instance, Connor explains: *“[The specific therapy or the therapists’ qualifications] didn’t matter to me. It was more about how they spoke to me and how they treated me that mattered.”* The most impactful practitioners were described as compassionate, patient, validating, and genuinely caring, with a willingness to sit with distress without rushing to fix it. Several spoke positively of professionals who adopted a trauma-informed stance, focusing on *“what happened to you”* rather than *“what’s wrong with you”*, which made participants feel understood rather than pathologized. In contrast, cold or clinical interactions, however technically skilled, were often experienced as distancing or re-traumatising. This highlights that for individuals with complex emotional and relational histories, the way care is delivered is just as important as the content of the intervention, and that trust, safety, and attunement are essential foundations for effective therapeutic work.

### Making sense of recovery: identity, stigma and the search for self

This superordinate theme was shared by all 12 participants and highlights the re-claiming of sense of self, and developing autonomy and self-compassion. It is depicted in three sub-themes, presented below.

#### “Who am I without my Disorder?”

Participants described the process of recovery as deeply entangled with questions of identity, often provoking a sense of confusion, fear, and loss. For many, both BN and BPD had become central components of how they understood themselves, particularly in the absence of stable self-concepts. The process of relinquishing disordered behaviours, even when actively desired, was accompanied by profound existential anxieties. Participants questioned who they would be without the disorders, how others might respond to them, and how they would manage the broader demands of the world, as Rebecca shares: *“Who am I without these illnesses? These disorders? And diagnoses? Like, how will people treat me if I don’t have them?”*. Recovery was therefore experienced as a paradoxical endeavour, in which a strong motivation to heal coexisted with an equally powerful fear of losing a core sense of self that had become central to everyday functioning and interpersonal relationships. This was especially pronounced for those with BPD, where unstable self-image is a core feature, and where BN behaviours often served as both a coping mechanism and identity anchor.

“I felt like I did want to get better (….) Every time something felt too much, like, if someone hurt me, or if I was, like, struggling with past experiences, I just turned everything, like, inward. It was like, hyper fixating on my body lessened the pain” (Leyla)

For six participants, receiving a diagnosis provided a framework for making sense of their emotional experiences and behavioural patterns. While the diagnostic label facilitated self-understanding, participants simultaneously positioned themselves as distinct from the disorders, using the diagnosis to externalise their difficulties. In this way, the diagnosis was experienced as clarifying both the origins and maintenance of their struggles, as well as the forms of support needed to navigate recovery. For several, the receipt of a diagnosis also fostered a greater sense of hope, with recovery perceived as more attainable.

“At first I was really taken aback by the diagnosis (….) Then I was like, ‘ah, ok, this all makes sense now” (Emalia)

“If it’s an illness, I can get better from it. Like, it’s not me (…) It’s like if I break my arm (…) somethings happened, I’m injured, I’ll get better” (Vienna)

In contrast, others described the diagnosis, especially of BPD, as disorienting, isolating, and stigmatising. Both external and internalised stigma emerged as pervasive concerns, with such experiences reported most prominently in relation to BPD. Internalised stigma reinforced self-critical narratives, while anticipated or actual external judgement from others contributed to social withdrawal and reluctance to seek support. Participants shared experiences of being labelled as manipulative, attention-seeking, or difficult, sometimes by professionals within the healthcare system, including in cases where the participant themselves worked in healthcare.

“I work in healthcare now and sometimes, colleagues read patient records and say like ‘they’ve got BPD. This will be horrific.’ And I’m just thinking ‘little do you know. I’ve got that’. And that’s really harmful and devastating for me to hear” (Hayley)

For five participants, recovery was experienced as a double bind: while there was a desire to move beyond the constraints of the disorders, the stigma attached to the diagnoses could drive further reliance on disordered behaviours as a means of coping with, or expressing, the very shame evoked by the diagnostic process. Others described using these behaviours as a form of self-punishment, internalising the negative narratives attached to their diagnoses.

“It’s like, oh yeah, you’ve been through all this shit, and that’s really terrible, and it’s not your fault. But you’ve got PD and you’re terrible and horrible (…) I felt like I needed punishing for being PD. But if people found out I was punishing myself I was ‘more PD’. It’s actually really f***ing sh**” (Georgia)

These encounters left a lasting imprint, shaping how individuals viewed their worthiness of care and their potential for recovery. These dynamics not only intensified participants’ emotional distress but also shaped their engagement with recovery, as the burden of shame frequently undermined self-worth and created ambivalence about change, as expressed by Kacey:

“For me, the diagnoses and the stigma just reinforced everything I thought about myself already. Terrible, horrible person, unlovable, doesn’t deserve recovery.”

In this way, recovery was experienced not only as a process of managing symptoms but also as a struggle to resist the negative identities imposed through diagnosis. This highlights how recovery, for many, is not just about behavioural change, but about rebuilding a fragmented sense of self in the aftermath of stigma, trauma, and chronic invalidation.

#### Recovery as relationship with self

A powerful and emergent theme in participants’ narratives was the gradual development of a more compassionate relationship with the self, often described as a significant and unfamiliar shift. For 10 participants, recovery involved the unlearning of internalised shame, self-criticism, and punitive coping mechanisms that had long shaped their responses to distress. Emery highlights this:

“I really had to unlearn the identity forced on me by others. Like, unlearning the ‘not good enough’, the ‘unlovable’, the ‘useless’. That’s hard when you’ve been told all of that for most of your life.”

Participants reflected on how, over time, they began to challenge harsh internal dialogues and replace them with more stable, accepting, and nurturing self-perceptions, often as a result of meaningful and validating therapeutic relationships. A consistent theme was that internalising the compassion shown by a key professional, whether a therapist,

support worker, or nurse, enabled participants to reframe how they related to themselves, fostering a sense of worthiness, capability, and hope.

“It was like I was recovering the ‘me’ that I could have been, should have been, if all my past traumas hadn’t have happened” (Georgia)

This transformation extended outward; as participants began to feel better about themselves, they described investing more into others, forming healthier and more reciprocal relationships, and contributing to something closely connected with their core values. For some, this took the form of working in healthcare, supporting friends and family, or participating in advocacy or research; actions that reinforced a sense of agency, purpose, and belonging.

“I just started to think that maybe I was capable and deserving of something more than my illnesses. Like, I wanted to use my difficulties for the good, and use it to help someone else” (Kacey)

Importantly, participants acknowledged that a positive self-relationship was not easily achieved and often involved a process of actively challenging deeply rooted beliefs formed through trauma or invalidating environments. Many were still navigating the tensions between old narratives of unworthiness and emerging feelings of self-respect and compassion. Nevertheless, this reorientation towards treating oneself with kindness and believing in one’s own value, was seen not only as a marker of recovery, but as its very foundation.

#### Recovery as reclaiming agency

Participants frequently described recovery as an act of reclaiming power, not only over BN and BPD, but over the narratives, relationships, and systems that had once defined or limited them. This shift from a passive experience of illness to an active pursuit of meaning, autonomy, and healing, marked a profound turning point, as shared by Kacey:

“I needed to feel like I was reclaiming my strength. Reclaiming my life. Like, the things I’ve been through will never have not happened, but I needed to know that it wasn’t controlling me and limiting me anymore.”

For all 12 participants, recovery meant choosing something better, not simply the absence of symptoms, but the presence of purpose, self-worth, and direction. This change was often experienced as reclaiming control, but in a different form: not control over the body, but over one’s choices, emotions, and future.

“I chose to live instead of just exist. I can do so much more than the constraints of my illnesses allowed. I started to know that I can do this” (Leyla)

Crucially, participants acknowledged that trauma, emotional sensitivity, or disordered thoughts had not disappeared, but they no longer dictated identity, relationships, or possibility. Instead of striving to eliminate distress entirely, recovery was framed as the capacity to live with it, without being consumed by it. Participants spoke of developing a sense of purpose, whether through relationships, meaningful work, or personal goals, which gave them reasons to choose recovery even on difficult days. However, a recurring tension emerged between participants’ internal goals and the focus of services they engaged with. Eleven participants described how treatment often centred on external behavioural changes such as eating regularly or stabilising weight, without adequately addressing the underlying causes, such as trauma, relational pain, or unmet emotional needs. This disconnection left participants feeling misunderstood and unsupported, reinforcing earlier experiences of powerlessness and emotional neglect. For example, Vienna expresses:

“It’s really hard to let go of a coping strategy that has literally been your only source of survival. And it’s even harder when the service you’re under only wants to focus on that rather than dealing with the issue that’s causing the coping strategy.”

Similarly, Sarah expressed that services unintentionally replicated the dynamics that contributed to her distress: *“They wanted me to be less emotional, less messy, but no one asked why I was like this”.* For these individuals, true recovery was not imposed from outside but chosen from within; a deeply personal commitment to seek something more meaningful, more authentic, and more sustainable than the illness had ever offered.

## Discussion

This study was the first to have explored the lived experiences of recovery from combined BN and BPD among adult individuals in the UK. The analysis revealed a deeply interconnected and non-linear experience of recovery, with themes such as trauma, identity, complex emotions, and relational dynamics frequently overlapping within participants’ narratives. Participants' descriptions of recovery as a deeply interconnected and non-linear process align with qualitative eating disorder recovery literature, which has consistently characterised recovery as involving periods of progress, setback, uncertainty, and ongoing identity negotiation rather than a fixed endpoint [23, 24]. However, participants in the present study described an additional layer of complexity, whereby reductions in BN symptoms often exposed ongoing difficulties associated with emotional dysregulation, interpersonal sensitivity, and identity disturbance linked to BPD. This suggests that recovery may be experienced as particularly fragile when eating disorder symptoms have functioned as a means of managing co-occurring psychological difficulties. Participants experienced recovery as a deeply identity-shaping process, often accompanied by confusion, fear, and a sense of loss. Both BN and BPD had become central to how individuals understood themselves, especially in the absence of a stable self-concept. This finding aligns with Loxton and Gleaves [8], who identified that BPD features are connected to eating disorder psychopathology via an affective disturbance domain, marked by chronic feelings of emptiness and disrupted self-concept. Furthermore, maladaptive behaviours were associated with abandonment avoidance, interpersonal difficulties, and ongoing identity disturbances. These results emphasize the critical role of identity disturbances within the combination of BN and BPD, highlighting the necessity for treatment interventions that specifically target identity-related and emotional regulation difficulties in this population. The blurring of boundaries between BN and BPD described by participants is consistent with theoretical accounts suggesting that eating disorder behaviours may serve important emotion-regulatory and interpersonal functions [8, 20]. From this perspective, bingeing and purging may be understood not solely as eating disorder symptoms, but as attempts to manage overwhelming affect, self-criticism, and relational distress. The present findings suggest that these functions may be particularly salient when BN co-occurs with BPD, where emotional dysregulation and identity disturbance are central features.

Letting go of disordered behaviours raised existential questions about who they were without these coping mechanisms. While some found relief or clarity through diagnosis, others found it isolating or stigmatising, especially when little post-diagnosis support or psychoeducation was provided. This further supports qualitative eating disorder recovery literature, which has highlighted identity reconstruction as a central component of recovery and described the challenge of defining oneself beyond the eating disorder [19, 25]. However, participants in the present study frequently described navigating not only an eating disorder identity but also the instability and uncertainty associated with BPD, suggesting that identity reconstruction may be particularly complex when recovery involves disentangling oneself from two highly influential diagnostic identities. This may be particularly important given that identity disturbance is considered a core feature of BPD and is increasingly recognised as central to long-term recovery and psychosocial functioning [20]. Recovery therefore appeared to involve not only moving beyond an eating disorder identity but also developing a more coherent and stable sense of self in the context of longstanding identity-related difficulties. Most expressed frustration with treatment systems that prioritised behavioural change without addressing the deeper emotional or relational wounds driving their struggles. This frustration left participants feeling unseen and disempowered, with some suggesting that services mirrored the invalidation they had long experienced. Consistent with findings by de Vos et al. [19] and LaMarre et al. [23], participants emphasised the importance of being understood, validated, and supported by others throughout recovery. However, participants in the present study frequently described experiences of stigma associated with both BN and BPD, including feeling dismissed, misunderstood, or viewed through diagnostic stereotypes. These experiences appeared to undermine hope and trust in services, highlighting how recovery may be shaped by the intersection of multiple stigmatised diagnoses. These findings are consistent with literature documenting stigma towards individuals with eating disorders and those diagnosed with BPD [10, 16]. However, participants' accounts suggest that these experiences may be compounded when both diagnoses are present, potentially creating additional barriers to help-seeking, treatment engagement, and recovery.

These experiences shaped participants’ understanding and conceptualisation of recovery. Participants described recovery from BN and BPD as an emotionally intense process, where letting go of disordered behaviours often magnified emotional vulnerability rather than alleviating it. Without the buffering effect of BN, many felt raw and exposed, struggling with lingering emotional dysregulation associated with BPD. This created a paradox: recovery brought hope, yet it also reopened wounds and intensified emotional chaos. For some, recovery felt like giving up the only method of control they had known in an unpredictable world. BN had served as a structure amidst internal instability, and letting go left many participants feeling fragile and uncertain. This finding is consistent with Robinson et al. [30], who describe an internal conflict contributing to ambivalence toward recovery, as individuals grapple with the loss of a familiar part of themselves and uncertainty about their ability to cope without the disorder. Importantly, participants did not describe BN and BPD as separate recovery processes. Rather, BN behaviours were frequently understood as serving a regulatory function in response to the emotional instability, interpersonal sensitivity, and identity disturbance associated with BPD [20]. Consequently, recovery from BN often exposed participants to difficulties that persisted beyond behavioural change, suggesting that symptom improvement in one condition may create new challenges when co-occurring difficulties remain. This finding builds upon existing qualitative eating disorder recovery literature, which has conceptualised recovery as a non-linear process involving identity reconstruction, emotional growth, and the development of a life beyond the eating disorder [19, 23, 24]. While this literature has highlighted the challenges associated with relinquishing eating disorder behaviours and rebuilding a sense of self, it has paid comparatively little attention to how recovery may be shaped by co-occurring conditions such as BPD. Participants' accounts suggest that recovery from BN can expose ongoing difficulties associated with emotional dysregulation, interpersonal sensitivity, and identity disturbance, creating challenges that extend beyond the recovery processes typically described within the eating disorder literature.

Additionally, recovery was understood as cyclical and fluctuating rather than a destination. Some participants rejected the idea of a fully “recovered” state and instead spoke of learning to maintain progress and manage setbacks. This required ongoing negotiation between a desire to heal and the fear of overwhelming emotional experiences. This is in alignment with Miller et al. [31, 32], who’s research reflects internal conflict and the importance of addressing identity disturbances and emotional regulation difficulties within treatment provisions. Central to this was the concept of control; many had used disordered behaviours as a way to manage internal and external chaos, and the transition away from those behaviours left a vacuum that was not easily filled without adequate emotional support.

Lastly, participants understood recovery as a deeply personal and evolving relationship with the self; one that often required unlearning years of self-criticism, shame, and internalised stigma. Many participants mentioned developing self-compassion as both a surprising and transformative part of recovery, learning to treat themselves with the kindness and belief they experienced through validating therapeutic relationships. This aligns with recent research emphasizing that recovery involves expanding one’s sense of self and fostering alignment with personal values; achieving this requires continuous self-reflection and the deliberate development of self-compassion [23]. This shift involved moving from harsh inner dialogues to more nurturing and supportive self-perceptions. Participants credited this change to internalising the care and belief of trusted professionals, which allowed them to begin seeing themselves as worthy of support and capable of change.

In summary, this study underscores the importance of compassionate, relationally attuned, and trauma-informed approaches to understanding and supporting recovery from combined BN and BPD. Recovery is not a linear or uniform process, nor can it be meaningfully reduced to symptom remission. Instead, it emerges as a complex, ongoing negotiation of identity, self-worth, emotional regulation, and connection; requiring care models that honour the lived realities and relational needs of those navigating it. Importantly, the findings suggest that recovery from BN and BPD may be best understood as an integrated process rather than as recovery from two separate disorders, highlighting the need for conceptual and clinical models that account for the reciprocal relationship between eating disorder symptoms, emotional regulation, and identity disturbance.

### Implications for clinical practice

These findings contribute to a growing body of research challenging symptom-focused models of recovery. Consistent with prior literature, recovery was experienced as cyclical and non-linear, shaped by trauma and grounded in relational processes such as validation, trust, and belonging. A key contribution of this study is the blurring of boundaries between BN and BPD, both diagnostically and experientially. Participants described shared mechanisms, emotional dysregulation, self-punishment, and identity instability, cutting across both conditions, highlighting the limitations of siloed diagnostic frameworks and the need for integrated, trauma-informed care.

Recovery was also marked by ambivalence, particularly the fear of losing coping strategies that had once provided stability. BN and BPD behaviours were often understood as attempts to manage overwhelming emotional states, underscoring the importance of framing recovery not as symptom removal, but as the development of alternative, meaningful ways of coping and relating to oneself and others. Clinicians should therefore recognise that reductions in eating disorder behaviours may initially expose individuals to heightened emotional distress, interpersonal sensitivity, and identity-related challenges. Supporting individuals to anticipate these difficulties and develop alternative coping strategies may help reduce the vulnerability that can accompany recovery. This supports recovery-oriented approaches that prioritise emotional safety, therapeutic relationships, and identity reconstruction over behavioural compliance alone.

The findings also highlight the importance of explicitly addressing identity within treatment. Supporting individuals to develop a sense of self beyond both BN and BPD may represent a key component of sustained recovery. Given participants' experiences of stigma and invalidation, clinicians should also be mindful of how diagnostic labels may shape therapeutic encounters and actively foster experiences of validation, understanding, and collaborative care.

Finally, systemic shortcomings in service access and continuity were evident. Participants described rejected referrals and premature discharge based on weight or perceived progress, often reinforcing shame and delaying engagement. These findings highlight the need to re-evaluate service thresholds and outcome measures to better reflect the complexity and risk associated with co-occurring BN and BPD. Greater collaboration between eating disorder and personality disorder services may also help address the fragmented care pathways described by participants and support more integrated approaches to treatment and recovery.

### Limitations and recommendation for future research

These findings should be interpreted in light of several limitations. First, the clinical characterisation of the sample was relatively limited. Although participants reported a previous diagnosis of BN and either a formal diagnosis of or self-identification with BPD, diagnostic status was based on participant report and was not independently verified through clinical records. Furthermore, detailed clinical information, including illness duration, symptom severity, treatment history, treatment modality, and number of previous treatment episodes, was not systematically collected. These factors are likely to influence how individuals experience and make sense of recovery, including the challenges they encounter and the aspects of treatment they perceive as most influential. In addition, including participants who either reported a formal diagnosis of BPD or self-identified as having BPD may have introduced undesired heterogeneity into the sample. From an IPA perspective, our aim was to recruit individuals who shared the lived experience of recovery from BN alongside BPD-related difficulties, rather than to establish diagnostically homogeneous groups in a psychiatric sense. Nevertheless, participants who self-identified with BPD may have differed in important ways from those with a formal diagnosis. Consequently, the findings should be interpreted as reflecting the recovery experiences of individuals who identified their difficulties as encompassing both BN and BPD-related experiences, rather than being assumed to apply exclusively to clinically verified BPD populations. Future qualitative research would benefit from collecting more comprehensive clinical information and examining whether recovery experiences differ according to diagnostic status and other clinical characteristics.

Second, the sample was recruited exclusively through a UK charity, introducing potential sampling bias. Participants, as peer support workers or ambassadors, may have been more reflective, motivated, and supported than the broader population of individuals recovering from co-occurring BN and BPD. Their access to training, resources, and structured peer support roles may have shaped their recovery experiences in ways that differ from individuals who are more isolated or at earlier stages of recovery. The overlap between personal and professional identities may also have influenced how participants reflected upon and articulated their experiences. Furthermore, participants were actively engaged in peer support, advocacy, or ambassadorial roles within the eating disorder sector and may therefore have been more accustomed to discussing their recovery than individuals who are not involved in such activities. Their continued engagement with recovery communities may also have influenced how they understood and narrated recovery, identity, and meaning-making. Consequently, these findings may not fully reflect the experiences of individuals recovering from co-occurring BN and BPD who are not connected to peer support networks or the wider eating disorder field.

Third, the self-selecting nature of the sample may over-represent individuals with more challenging or traumatic recovery experiences, while underrepresenting those with more positive or less complex trajectories. This raises the possibility that certain recovery narratives remain underexplored.

Fourth, the sample may not reflect the diversity of individuals recovering from co-occurring BN and BPD, particularly with respect to gender, ethnicity, cultural background, and socioeconomic circumstances. As such, the findings may not capture the full range of intersecting experiences within this population. Future research should prioritise more diverse and representative samples, with greater attention to intersectionality and systemic influences. Longitudinal and mixed-methods approaches could further illuminate recovery trajectories over time, while additional qualitative work could deepen understanding of the relational, cultural, and structural contexts shaping recovery.

## Conclusion

This study highlights that recovery from co-occurring BN and BPD is not a linear endpoint, but a deeply personal and often fragile process shaped by identity, trauma, and emotional regulation. Central to this journey are relational experiences: validation, trust, and belonging, all of which underpin meaningful change and emphasise the need for compassionate, attuned therapeutic alliances. Importantly, participants described a blurring of boundaries between BN and BPD, pointing to shared underlying mechanisms rather than distinct conditions. As such, these findings challenge siloed diagnostic approaches and strengthen the case for integrated, trauma-informed care. Taken together, these findings call for a shift in how recovery is understood and supported: not as symptom reduction alone, but as the reconstruction of self, relationships, and meaning. This necessitates rethinking current service models and eligibility criteria, particularly where they overlook the underlying trauma and emotional distress sustaining these conditions.

## Data Availability

The datasets used and/or analysed during the current study are available from the corresponding author on reasonable request.

## Abbreviations

BN: Bulimia nervosa
BPD: Borderline personality disorder
CBT: Cognitive behavioural therapy
DBT: Dialectical behavioural therapy
IPA: Interpretative phenomenological analysis
